# Modeling the Effects of Policies that Restrict Tobacco Retail Outlets on Prenatal Smoke Exposure and Perinatal Health Care Utilization

**DOI:** 10.1007/s11121-024-01718-2

**Published:** 2024-09-04

**Authors:** Joseph Boyle, D. Jeremy Barsell, Junfeng Jim Zhang, Jason A. Oliver, F. Joseph McClernon, Bassam Dahman, Cathrine Hoyo, Bernard F. Fuemmeler, David C. Wheeler

**Affiliations:** 1 Department of Family Medicine and Population Health, School of Medicine, Virginia Commonwealth University, Richmond, VA 23298 USA; 2 Environmental Science and Policy Division, Duke Global Health Institute and Nicholas School of the Environment, Durham, NC 27708 USA; 3 Department of Family and Preventive Medicine, University of Oklahoma Health Sciences Center, Oklahoma City, OK 73104 USA; 4 Department of Psychiatry and Behavioral Sciences, Duke University School of Medicine, Durham, NC 27705 USA; 5 Department of Social Behavioral Science, School of Population Health, Virginia Commonwealth University, Richmond, VA 23298 USA; 6 Department of Biological Sciences, Center for Human Health and the Environment, North Carolina State University, Raleigh, NC 27695 USA; 7 Department of Biostatistics, School of Population Health, Virginia Commonwealth University, Richmond, VA 23298 USA

## Abstract

Tobacco retail outlet (TRO) density has been associated with increased cotinine levels in pregnant persons and their children. As such, the higher densities of TROs may represent higher levels of active smoking during pregnancy. The purpose of this study is to simulate the reduction in cotinine (a biomarker of smoke exposure) and health care utilization that could occur in pregnant persons under enactment of several candidate TRO reduction policy recommendations. Using existing retail outlet data from the state of North Carolina and from the Newborn Epigenetic Study (NEST), the present study created hypothetical policy-informed datasets of TROs that a) limited the number of TROs to the same density as the 2014 San Francisco (SF) policy (Policy 1), b) set the minimum distance to 500 feet between TROs from a school and from other TROs (Policy 2), c) restricted the types of TROs to exclude pharmacies (Policy 3), and d) a combination of Policies 1–3 (Policy 4). We estimated the effects of each policy individually and in a separate model with their combined effects in terms of the reduction on cotinine levels and health care utilization, as measured by number of visits to the emergency department (ED). We found that the hypothetical policies were likely to be effective in reducing maternal cotinine and ED visits, with the majority of the mothers in the dataset demonstrating reductions in these outcomes after implementation of the policies. We found that Policy 1 led to moderate reductions in TRO exposure for the majority of the sample as well as stratified by race/ethnicity. Additionally, Policy 4 had slightly larger estimated effects than Policy 1, but could be more onerous to implement in practice. Overall, we identified evidence supporting the efficacy of TRO reduction strategies that could impact smoke exposure during pregnancy in our diverse sample in North Carolina.

**Supplementary Information:**

The online version contains supplementary material available at 10.1007/s11121-024-01718-2.

## Introduction

Tobacco retail outlet (TRO) density has been associated with increased cotinine levels in pregnant persons and their children (Wheeler et al., 2020, 2021). As such, the higher densities of TROs may represent higher levels of active smoking during pregnancy, as well as secondhand smoke (SHS) exposure (Wheeler et al., 2022a). Elevated levels of cotinine in pregnancy are associated with numerous adverse health effects for children (e.g., asthma, allergies, cancer, epigenetic changes) (Braun et al., 2020; Cosin-Tomas et al., 2022; Gollenberg et al., 2015; Lisboa et al., 2012; Fuemmeler et al., 2021), which in turn increases health care utilization (Fuemmeler et al., 2024). Reducing TRO density could serve as an effective public health intervention that would not only mitigate adverse health outcomes for pregnant persons and their children, but could also reduce associated health care costs.

In the past, policy efforts regarding TROs have been focused on reducing the supply of and demand for tobacco products. As outlined by the World Health Organization Framework Convention on Tobacco Control (WHO FCTC), efforts primarily focused on increasing taxes for the sale of tobacco products, banning advertising, reducing illicit sales (e.g., to minors), providing cessation resources, and promoting smoke free public spaces (WHO Framework Convention on Tobacco Control World Health Organization, 2003). It was not until more recently that policies shifted to reducing the number of TROs themselves. A recent scoping review conducted by Alebshehy et al. (2023) examined multiple different policies that were enacted to reduce the overall supply of tobacco. Of the various different types of tobacco control policies, those that directly regulated the retail environment tended to have strong effects in reducing tobacco use, compared to other policies that simply limited the sale of tobacco products (e.g., limiting hours or days that tobacco products can be sold). The review highlights a general finding that people would smoke less if they had to travel further to obtain tobacco products (Canada, 2005) and if the process of obtaining tobacco was more inconvenient. Notably, this would be a direct effect of reducing TRO density and proximity (Alebshehy et al., 2023).

A number of policy recommendations have been suggested and implemented in previous research that have the potential to restrict the number of TROs in order to decrease tobacco availability and to address health inequities across racial/ethnic minorities and those in lower socioeconomic areas (Ackerman et al., 2017; Kong et al., 2022; Mills et al., 2022). In San Francisco, a 2014 policy was enacted that capped the number of TROs to 45 per supervisorial district and prohibited new permits to retailers within 500 feet of school or other retailers (City of San Francisco C, 2014). While this policy was effective in decreasing TRO density across the city (Bright Research Group, 2016), simply reducing the number of TROs alone would not completely mitigate the existing disparities for racial/ethnic minorities and in lower socioeconomic areas, because TRO density is much higher in areas with greater percentage of Black and Hispanic/Latino residents and in areas with higher percentage of those living under the poverty line (Kong et al., 2021, 2022). Another policy recommendation to address these inequities includes restricting tobacco sales to certain retailer types (Mills et al., 2022; Lee et al., 2022). Tobacco is sold in many different types of retailers, such as grocery stores, convenience stores, and pharmacies. Banning tobacco sales in pharmacies would further reduce TRO density, as shown to be effective in California and Massachusetts (Jin et al., 2016). Furthermore, limiting TRO exposures may engender lower rates of tobacco initiation, as one cross-sectional study found that lower TRO density was associated with a correspondingly lower likelihood of smoking initiation in young adults (Cantrell et al., 2016). Similar opportunities also arise for cessation, as one study conducted across the United States found that lower TRO density proximate to current smokers’ residence was associated with greater odds of 30-day smoking abstinence (Cantrell et al., 2015).

The purpose of this study is to simulate the reduction in cotinine (a biomarker of smoke exposure) and health care utilization that could occur in pregnant persons under enactment of several candidate TRO reduction policy recommendations. Given that these policies have been tested in only a few real-world settings, modeling these effects will inform policy makers of the most effective policy or combination of policies to reduce the adverse effects of TROs in a given community. Using existing retail outlet data from the state of North Carolina and from the Newborn Epigenetic Study (NEST), the present study created hypothetical policy-informed datasets of TROs that a) limited the number of TROs to the same density as the 2014 San Francisco (SF) policy, b) set the minimum distance to 500 feet between TROs from a school and from other TROs, and c) restricted the types of TROs to exclude pharmacies, because pharmacies typically dispense medication to improve health but also at times sell tobacco products (Seidenberg et al., 2013; Wang et al., 2016). We estimated the effects of each policy individually and in a separate model with their combined effects in terms of the reduction on cotinine levels and health care utilization, as measured by number of visits to the emergency department (ED).

## Methods

### Study Sample

The *Newborn Epigenetics Study* (NEST) was a prospective cohort study that was designed to investigate how certain maternal behaviors (e.g., nutrition, smoking, and other exposures) may influence the environment in utero, which in turn influences the epigenome, birth weight of children, as well as early childhood growth and health. Study enrollment for NEST occurred between 2005 and 2011. During this period, 2,595 pregnant persons consented and enrolled during their first prenatal clinic visit at one of six prenatal clinics in Durham County, North Carolina (NC), during which survey data and maternal blood specimens were obtained. Of those consenting and enrolling, cotinine levels were measured. Recruitment occurred in two waves, with criteria for the first wave (n = 853) including those with singleton births who spoke English, had available prenatal blood sampled, and who consented to future research. The second wave (n = 288) had expanded eligibility criteria including residing in Durham County at the time of enrollment with available prenatal blood samples and who had declined further follow-up as part of the parent NEST study. In our analysis, we included pregnant persons who resided in Durham County and the adjacent five counties (Chatham, Granville, Orange, Person, and Wake) at the time of enrollment, had available prenatal cotinine blood measures, and covariate data (n = 1,055). Durham and Wake counties are predominately urban and contain the major cities of Durham and Raleigh, respectively. Orange County is partly urban, containing the city of Chapel Hill, and partly rural; Chatham, Granville, and Person counties are predominately rural.

### Smoke Exposure

The level of smoke exposure was determined with cotinine assays from prenatal maternal plasma simples. Liquid chromatography-mass spectrometry (LC–MS/MS) measured cotinine levels using a detection limit of 0.05 ng/mL. The LC–MS/MS system consists of a ThermoFinnigan TSQ Quantum Ultra triple-stage quadrupole mass spectrometer containing atmospheric pressure chemical ionization and electrospray ionization sources and is coupled to an Agilent 1200 liquid chromatograph.

### Tobacco Retail Outlets (TROs)

The process that we used to create a database of likely TROs in our study region has been described previously (Wheeler et al., 2020, 2022a, 2022b). Briefly, we used the National Establishment Time Series (NETS) dataset, a record of business types in the United States ranging from 2000 to 2019, to begin with an initial set of possible TROs in our study region. We then applied an algorithm that filtered the businesses using their North American Industry Classification System (NAICS) codes to identify probable TROs. Then, we manually verified the existence of these TROs using the archive feature in Google Street View (GSV) to locate the business. We additionally verified TRO locations in Durham County using a database from the University of North Carolina at Chapel Hill and Duke University, produced by researchers ground-truthing TROs (Oliver et al., 2021). These processes constituted an extensive effort to attain a definite conclusion for any potential TRO in the study region. Overall, we confirmed greater than 99 percent of active TROs in our database.

### Statistical Models

For each outcome variable, we fit the following statistical model:

$$f\left({y}_{i}\right)={\beta }_{0}+{\beta }_{1}*TR{O}_{i}+{\sum }_{b=1}^{B}{\theta }_{b}{x}_{ib}$$

Here, ${y}_{i}$ is the outcome variable for the ${i}^{th}$ mother in the dataset with either log cotinine or count of visits to the ED. The function $f\left(\cdot \right)$ is dependent on the nature of the outcome variable. For the continuous cotinine outcome, we used the identity function and modeled the log cotinine value directly, meaning that the model is a linear regression model. For the count of ED visits outcome, we modeled the logarithm of the count, using negative binomial regression models (which we used in contrast to Poisson regression models owing to overdispersion in the outcome variable). In both cases, ${\beta }_{0}$ represents an intercept term, and we adjusted for relevant patient-level covariates ${x}_{ib}$ with regression coefficients ${\uptheta }_{b}$. Specifically, we adjusted for age, race/ethnicity (White, Black, Hispanic, or other/missing), education level (less than high school, high school graduate/GED, some college, college graduate, missing), and marital status (never married, married, living with partner, divorced/separated, other, missing). We have used these covariates in previous analyses owing to associations with maternal cotinine, such that each of these covariates acts as a confounder for cotinine levels among individuals in this dataset (Wheeler et al., 2020, 2022a). By adjusting for these covariates, we improve precision in the marginal estimates of the policies’ effect on cotinine levels. Finally, ${\upbeta }_{1}$ is the coefficient for $TR{O}_{i}$, the primary exposure variable. We calculated this variable by counting the number of TROs located within a two-mile radius of the mother’s residence, and we have used this variable in previous analyses because it best explained variation in maternal cotinine values (Wheeler et al., 2022a).

### Policies

There are four policies that we used to evaluate the number of TROs. In Policy 1, we limited the number of TROs to the same density as the city of SF implemented in 2014. SF consists of 11 Supervisorial Districts, and the city’s restriction of 45 TROs per district is approximately equivalent to 1 TRO per 1700 people (calculations provided in Supplemental Material Appendix S1). Because our study region consists of 6 counties with varying population sizes, we implemented Policy 1 on a county-wide basis, restricting the number of TROs in that county to the same density enacted in the SF policy, based on the county population during the study period. This involved removing TROs from the dataset in each county. However, since there are many ways to remove a number of TROs from the set operating in one county, and since different ways of doing so would result in different exposure values for the pregnant persons (different numbers within two miles of their residence), we created 100 hypothetical datasets for this policy that achieved the same density as the SF policy. We created many hypothetical datasets in order to capture the uncertainty associated with the TRO removal in this policy, and we used all datasets to estimate the policy’s effect.

In Policy 2, we set a minimum distance of 500 feet between TROs and a school and between TROs and other TROs. For the first component of this policy, we obtained a list of all public schools in our study region and geocoded their addresses. Then, we computed the straight-line distance between all schools and all TROs in the dataset, and removed any TROs falling within 500 feet of a school. Then, for the second component of the policy, we used an iterative algorithm to achieve the policy constraint. Specifically, we computed the distance matrix between all TROs. Then, among the set of pairs of TROs falling within 500 feet of each other, we randomly chose 1 TRO to remove from the dataset. We then re-computed the distance matrix between TROs and repeated the process until no TRO in the dataset was within 500 feet of another TRO. We also created 100 hypothetical datasets for this policy to account for uncertainty in the stochastic removal of TROs.

In Policy 3, we restricted the types of TROs to exclude pharmacies. This policy was deterministic, and therefore we only needed to create one dataset that removed any TRO whose NAICS code indicated that it was a pharmacy or that had the word “pharmacy” in their company name.

In Policy 4, we implemented all of the preceding policies in combination. First, we removed all pharmacies from the dataset as in Policy 3. Then, we reduced the density of TROs in each county to that implemented by the SF policy (Policy 1). Then, we removed remaining TROs as in Policy 2 that were within 500 feet of a school or another TRO. This process resulted in 100 hypothetical datasets.

For each hypothetical dataset created in this process, we calculated the number of TROs in the dataset within two miles of each mother in our data. Therefore, the ${i}^{th}$ mother had primary exposure values $TR{O}_{i}$, for the real (observed) count falling within two miles of her residence; the set $\{TR{O}_{i,\text{1,1}},\dots ,TR{O}_{i,\text{1,100}}\}$ brought about by Policy 1; the set $\{TR{O}_{i,\text{2,1}},\dots ,TR{O}_{i,\text{2,100}}\}$ brought about by Policy 2; $TR{O}_{i,\text{3,1}}$ brought about by Policy 3, and the set $\{TR{O}_{i,\text{4,1}},\dots ,TR{O}_{i,\text{4,100}}\}$ brought about by Policy 4.

### Inference

For each outcome (cotinine and ED visits), we saved the regression coefficient${\upbeta }_{1}$, which estimates the association between the TRO exposure and the outcome after adjusting for the covariates. Then, for each hypothetical dataset $k$ created by policy $j$ for each mother$i$, we estimated the reduction in the outcome variable associated with the TRO reduction caused by the policy, using the expression ${\upbeta }_{1}\left(TR{O}_{i}-TR{O}_{i,j,k}\right)$. Then, we summarized the effect of the policy by exponentiating the average of these values (because the cotinine analyses model the logarithm of the cotinine values in linear regression and the ED analyses model the logarithm of the number of visits in negative binomial regression) over all datasets for each mother and then averaging over all pregnant persons in the dataset. We additionally stratified these summaries by race/ethnicity. For computations, we fit all models in R Version 4.3.1 and used a level of α = 0.05 for statistical significance.

## Results

The summaries of key variables for the pregnant persons in the analysis dataset are displayed in Table 1. The summaries of: a) TRO reduction for Policies 1–4 are displayed in Table 2; b) cotinine reduction for Policies 1–4 are displayed in Table 3; and c) healthcare utilization for Policies 1–4 are displayed in Table 4. For the cotinine outcome, after adjusting for race, age, education level, and marital status, the number of TROs within two miles of a mother’s residence was positively and significantly associated with maternal cotinine level. A one-unit increase in this exposure variable was associated with an increase of 1.007 ng/mL cotinine. Subsequently, we applied the four candidate policies to our sample. A summary of the number of TROs in the resulting hypothetical datasets is given in Supplemental Material Table S1 and shows that Policies 1 and 2 reduced the overall prevalence of TROs in the study region by approximately a third, Policy 3 had little effect on the number of TROs, and Policy 4 had a slightly larger effect by combining all of the three previous policies.

**Table 1:** Summary of sample characteristics (n = 1055)

Variable	Value
Age (years)	27.48 (5.77)
Cotinine (ng/mL) ^a^	0.68 [0.25, 2.52]
Emergency Department visits	2.57 (3.84)
Race	
Black	540 (51.2)
Hispanic	100 (9.5)
Other/Missing	53 (5.0)
White	362 (34.3)
Education level	
College graduate	364 (34.5)
Some college	208 (19.7)
High school graduate/GED	205 (19.4)
Less than high school	165 (15.6)
Missing	113 (10.7)
Marital status	
Never married	291 (27.6)
Married	419 (39.7)
Living with partner	179 (17.0)
Divorced/separated	32 (3.0)
Other	18 (1.7)
Missing	116 (11.0)

Continuous values summarized with mean and standard deviation unless otherwise indicated. Categorical variables summarized with count and percent

^a^ Summarized with median and (25th percentile, 75th percentile) due to skewness

**Table 2:** Summary of TRO reductions for Policies 1–4 overall and by race/ethnic groups

Group	Policy 1		Policy 2		Policy 3		Policy 4	
	Median (95% interval)	[25th, 75th] percentile	Median (95% interval)	[25th, 75th] percentile	Median (95% interval)	[25th, 75th] percentile	Median (95% interval)	[25th, 75th] percentile
Overall	19.60 (0, 79.57)	[7.00, 42.98]	8.87 (0, 45.66)	[0, 21.27]	0 (0, 9.00)	[0, 0]	22.20 (0, 87.63)	[7.82, 48.37]
White	7.34 (0, 70.52)	[0.11, 16.57]	0 (0, 37.51)	[0, 8.85]	0 (0, 2.00)	[0, 0]	8.76 (0, 77.6)	[0.36, 19.40]
Black	30.76 (0, 80.26)	[13.61, 53.86]	14.74 (0, 46.59)	[3.50, 27.76]	0 (0, 10.53)	[0, 0]	35.52 (0, 88.20)	[15.06, 59.21]
Hispanic	32.94 (0.19, 79.77)	[13.49, 53.29]	16.33 (0, 46.90)	[5.19, 29.63]	0 (0, 15.00)	[0, 0]	37.85 (0.46, 87.88)	[17.03, 57.44]
Other	20.83 (0, 78.84)	[11.55, 36.13]	8.87 (0, 45.36)	[3.04, 15.70]	0 (0, 6.80)	[0, 0]	23.45 (0, 86.68)	[13.66, 42.67]

TRO reduction with policy denotes the distribution of reductions in the exposure variable (TROs within 2 miles)

**Table 3:** Summary of cotinine reduction outcome for Policies 1–4 overall and by race/ethnicity

Group	Policy 1		Policy 2		Policy 3		Policy 4	
	Median (95% interval)	[25th, 75th] percentile	Median (95% interval)	[25th, 75th] percentile	Median (95% interval)	[25th, 75th] percentile	Median (95% interval)	[25th, 75th] percentile
Overall	1.14 (0, 1.69)	[1.05, 1.33]	1.06 (0, 1.35)	[0, 1.15]	0 (0, 1.06)	[0, 0]	1.16 (0, 1.78)	[1.05, 1.37]
White	1.05 (0, 1.59)	[0.11, 1.12]	0 (0, 1.28)	[0, 1.06]	0 (0, 1.01)	[0, 0]	1.06 (0, 1.67)	[0.29, 1.14]
Black	1.22 (0, 1.69)	[1.09, 1.42]	1.10 (0, 1.36)	[1.02, 1.20]	0 (0, 1.07)	[0, 0]	1.26 (0, 1.79)	[1.10, 1.48]
Hispanic	1.24 (0.15, 1.69)	[1.09, 1.42]	1.11 (0, 1.36)	[1.03, 1.21]	0 (0, 1.10)	[0, 0]	1.28 (0.30, 1.78)	[1.12, 1.46]
Other	1.15 (0, 1.68)	[1.08, 1.27]	1.06 (0, 1.35)	[1.02, 1.11]	0 (0, 1.05)	[0, 0]	1.17 (0, 1.77)	[1.09, 1.32]

Cotinine reduction with policy denotes the distribution of reductions in the outcome variable (cotinine; units of ng/mL) due to reductions in the exposure variable

**Table 4:** Summary of healthcare utilization outcome for Policies 1–4 overall and by race/ethnicity

Group	Policy 1		Policy 2		Policy 3		Policy 4	
	Median (95% interval)	[25th, 75th] percentile	Median (95% interval)	[25th, 75th] percentile	Median (95% interval)	[25th, 75th] percentile	Median (95% interval)	[25th, 75th] percentile
Overall	1.06 (0, 1.28)	[1.02, 1.14]	1.03 (0, 1.15)	[0, 1.07]	0 (0, 1.03)	[0, 0]	1.06 (0, 1.25)	[1.02, 1.13]
White	1.02 (0, 1.20)	[0, 1.05]	0 (0, 1.12)	[0, 1.03]	0 (0, 1.01)	[0, 0]	1.02 (0, 1.22)	[0, 1.05]
Black	1.10 (0, 1.23)	[1.04, 1.18]	1.05 (0, 1.16)	[1.01, 1.09]	0 (0, 1.03)	[0, 0]	1.09 (0, 1.25)	[1.04, 1.16]
Hispanic	1.11 (0.14, 1.21)	[1.05, 1.18]	1.05 (0, 1.15)	[1.02, 1.09]	0 (0, 1.05)	[0, 0]	1.10 (0, 1.22)	[1.05, 1.16]
Other	1.07 (0, 1.22)	[1.04, 1.12]	1.03 (0, 1.15)	[1.01, 1.05]	0 (0, 1.02)	[0, 0]	1.06 (0, 1.25)	[1.04, 1.11]

This table only summarizes the reductions in the ED visits outcome variable because the changes in the exposure variable are identical for each policy to what is shown in Tables 2, 3

The distribution of hypothetical effects of Policy 1 on the cotinine outcome is given in Table 3. A map illustrating the effect of Policy 1 on the TRO landscape with an example dataset is provided in Supplemental Material Figure S1. On average across our sample, this policy reduced the number of TROs within two miles of a mother’s residence by 19.6, with steeper reductions in exposure occurring for Black and Hispanic pregnant persons than for White pregnant persons (as shown in Table 2). This led to an average reduction of 1.14 ng/mL in cotinine. This policy led to cotinine reductions for more than 75 percent of the overall sample (the (25th, 75th) percentile of cotinine reduction was (1.05, 1.33)) and in each racial/ethnic subgroup.

Policy 2 led to a more modest reduction of 8.9 TROs in exposure, and an average reduction of 1.06 ng/mL in cotinine (Table 3). This policy reduced the median TRO and cotinine exposure for Black and Hispanic mothers but not for White mothers. Policy 3 that prohibited pharmacies from selling tobacco had an almost universally null effect on the TRO exposure and cotinine outcome (Tables 2 and 3), because very few TROs in the dataset were pharmacies. Policy 4 that combined the previous three policies had the largest effect, reducing the TRO exposure by 22.2 TROs and cotinine by 1.16 ng/mL (Tables 2 and 3). Similarly to the other policies, this policy saw larger improvements for Black and Hispanic mothers than White mothers in TRO reduction (35.5 and 37.9 vs. 8.8 TROs) and cotinine reduction (1.26 and 1.28 vs 1.06 ng/mL). As in Policy 1, Policy 4 led to a reduction in cotinine for more than 75 percent of mothers in the dataset overall and for all racial/ethnic subgroups.

Additionally, the TRO exposure variable was positively and significantly associated with the healthcare utilization outcome of the count of number of ED visits. Adjusting for covariates, a one-unit increase in the number of TROs within two miles of a mother’s residence was associated with an increase of 1.003 in the rate of ED visits. Of the candidate policies considered, Policy 1 (1.06 visits), Policy 2 (1.03 visits), and Policy 4 (1.06 visits) demonstrated a hypothetical cotinine reduction effect (Table 4). For each of these policies, the effect was larger for Black and Hispanic mothers than for White mothers. Additionally, Policy 1 and Policy 4 reduced cotinine values for all racial/ethnic subgroups.

## Discussion

In this paper, we estimated the efficacy of four hypothetical interventions to reduce the density of TROs in and around Durham County, North Carolina. We began by estimating the associations of a TRO exposure variable with two health outcomes (cotinine and number of ED visits) among a sample of mothers in this study region. By doing so, we were able to estimate the associated improvements in the health outcomes through reductions in the TRO environment for these mothers. Generally, we found that the hypothetical policies were likely to be effective in reducing maternal cotinine and ED visits, with the majority of the mothers in the dataset demonstrating reductions in these outcomes after implementation of the policies. Typical estimated reductions in cotinine associated with policies to limit TRO exposures were approximately 1 ng/mL. From purely a retail environment perspective, this reduction is considerable, given that a threshold of 3 ng/mL is commonly used to categorize non-active from active smokers (Benowitz et al., 2009). We found that Policy 1, which limited the density of TROs in each county to a threshold implemented by a policy enacted in the city of San Francisco, led to moderate to sizeable reductions in TRO exposure for the majority of the sample as well as stratified by race/ethnicity. Specifically, on average, Policies 1, 2, and 4 led to average reductions of 20, 9, and 22 TROs within a two-mile radius for participants in our sample, which corresponds to a notable reduction in availability of tobacco products. Other policies, such as one that restricted pharmacies from selling tobacco, had little effect in our models. Additionally, Policy 4 which combined separate policies frequently had slightly larger estimated effects than Policy 1, but could be more onerous to implement in practice. Overall, we identified evidence supporting the potential efficacy of TRO reduction strategies that could impact smoke exposure during pregnancy in our diverse sample in North Carolina.

The findings in this study compare similarly to the actual policies implemented in California (City of San Francisco C, 2014). If Policies 1 and 2 were implemented in this area of North Carolina, there would be significant reductions in TRO density, which would lead to reducing maternal cotinine and ED visits. Most notably, Policy 1, as described above, would also advance equity in tobacco control, given that TRO density is more concentrated in neighborhoods with lower SES and higher proportions of racial/ethnic minority groups (Kong et al., 2021, 2022).

In contrast to its real world counterparts in California and Massachusettes (Jin et al. 2016), Policy 3 was not effective, at least in our sample in North Carolina. Some literature has estimated that 10 percent of all tobacco retailers are pharmacies (Seidenberg et al. 2013; Tobacco Control Legal Consortium, 2018)], and that in North Carolina specifically, pharmacies were three times as likely to not comply with Family Smoking Prevention and Control Act regulations (Rose et al., 2013). Other studies have even recommended that banning tobacco sales at pharmacies would be more effective than reducing overall numbers of TROs (Luke et al., 2017). Per the results of our study, however, there is insufficient evidence that restricting tobacco sales at pharmacies would be more effective than other proposed policies. One reason for this outcome in our study was that very few TROs in our dataset were classified as pharmacies. This reflects the 2014 decision by CVS and similar pharmacies to discontinue selling tobacco products (Brennan & Schroeder, 2014); larger estimated effects of such policies in previous studies operated in a context where more pharmacies were selling tobacco products. Notably, there are some nuances here in the data that do not align with real-world circumstances, since some policies that restrict sale in pharmacies can exempt certain grocery stores that also contain a pharmacy. More research is needed in this area to address the impact of restricting tobacco sales in pharmacies, particularly in tobacco retailer landscapes where pharmacies play a larger role and in cases where there are grocery stores that contain pharmacies that are also selling tobacco products.

There are several strengths to our study. First, we implemented and compared a variety of hypothetical TRO reduction policies that were inspired by strategies posed in the literature or implemented in practice. Considering policies that are realistic given the current tobacco and regulatory landscapes is important in order to avoid analysis of tobacco control strategies that are not feasible. Comparing the effects of these hypothetical policies on a fixed analysis sample provides a point of comparison to identify which strategies are most effective. Additionally, we created 100 hypothetical datasets for most of the hypothetical strategies. This allowed us to account for the uncertainty inherent in reducing the number of TROs, such as which TROs would close under implementation of certain policies and how this could differentially affect the exposure variables for mothers in the dataset.

The strengths of our study should be viewed in the context of its limitations. First, we are only able to generalize the results of our study to our population of mothers in and around Durham County, North Carolina. The effects of these policies could differ for different demographic groups, regions of the country, or by other factors. However, our sample was reasonably diverse with respect to race, ethnicity, and educational attainment and therefore has a reasonable degree of representativeness of the United States population. Second, it is possible that our database of TROs in the study region did not exactly reflect the true TRO landscape. However, we made extensive efforts to verify all TROs in the dataset and used multiple data sources to identify possible TROs, so the degree of bias brought about by this factor is likely to be minimal. Third, we considered four policies (three unique ones and one combination of the three) to estimate their effects on tobacco-related health outcomes. However, it is possible that other strategies to reduce the number of TROs could have different and possible larger effects on these health outcomes.

In summary, we found that several of the proposed policies would be effective in reducing the density of TROs in Durham County and its surrounding counties in North Carolina. Through our modeling methods, we were able to estimate the associated improvements in two health outcomes through reductions in the TRO environment for these mothers. Furthermore, we found that the hypothetical policies were effective in reducing maternal cotinine and ED visits, with the majority of the mothers’ demonstrating reductions in these harmful outcomes after implementation of the policies. Out of the proposed policies, Policy 1 that explicitly limited TRO density led to moderate reductions in TRO exposure for the majority of the sample as well as stratified by race/ethnicity. In general, we identified evidence supporting the efficacy of TRO reduction strategies for mothers in North Carolina. In practical terms, focusing efforts to enact both Policies 1 and 2 would lead to the greatest reduction in TRO density as well as reductions in maternal cotinine and ED visits. These policies also enjoy the added benefit of having precedent in real-world applications, having been deployed successfully in certain areas. Of course, while the ideal exposure to tobacco products is none, these policies that limit density and proximity are valuable in limiting accessibility to the tobacco retail environment and offer meaningful progress in limiting pathways for exposure. These estimations are hypothetical, but worth pursuing for public health change. Future research in this area is needed to estimate the impact of restricting tobacco sales at pharmacies. Additionally, the methods in this study can be applied to other geographical locations in the United States, as the TRO landscape may differ from state to state.

## Supplementary Information

Below is the link to the electronic supplementary material.

Supplementary file1 (DOCX 50 KB)
