# Supplementary material for: Modeling the Effects of Policies that Restrict Tobacco Retail Outlets on Prenatal Smoke Exposure and Perinatal Health Care Utilization
Source: Prev Sci. 2024 Sep 4;25(6):910–8. doi: 10.1007/s11121-024-01718-2 (PMC11390818; doi:10.1007/s11121-024-01718-2)
Supplement: Supplementary file 1 — Supplementary file1 (DOCX 50 KB) [file 11121_2024_1718_MOESM1_ESM.docx]

**Supplemental Material**

**Modeling the Effects of Policies that Restrict Tobacco Retail Outlets on Prenatal Smoke Exposure and Perinatal Health Care Utilization**

Joseph Boyle, Ph.D., D. Jeremy Barsell, M.S., Bernard F. Fuemmeler. Ph.D., David C. Wheeler, Ph.D.

**Appendix S1.** Calculations to determine the equivalent density for counties in study region for Policy 1.

The policy enacted by the city of San Francisco (SF) in 2014 reduced the number of TROs to 45 per Supervisorial District (SD). There are 11 SDs in SF. The 2012-2016 American Community Survey 5-year estimates for the population comprising each SD are (79970, 68390, 72360, 77000, 84030, 69360, 74710, 68200, 86430, 75250, 86120) ^[[1]](#footnote-1)^. The mean value of this population vector divided by 45 is 1700. Therefore, in our implementation of Policy 1, we restricted the number of TROs for each county in our study region to be no more than 1 per 1700 residents.

**Table S1.** Summary of number of TROs in hypothetical policy datasets by policy.

| Policy | Minimum | Median | Maximum |
| --- | --- | --- | --- |
| 1 | 825 | 825 | 825 |
| 2 | 865 | 871 | 878 |
| 3 | 1258 | 1258 | 1258 |
| 4 | 622 | 631 | 643 |

**Note:** There were 1295 TROs in our study region prior to the introduction of any policies. Only one hypothetical dataset was necessary to enact Policy 3, which restricted the types of TROs to prohibit pharmacies. Also, 100 distinct datasets were created for Policy 1, but since all datasets limited the density of TROs per county to the same threshold, the number of TROs in the study region was constant over all datasets.

**Figure S1.** Illustration of TRO landscape in the study region before and after implementation of one hypothetical policy (Policy 1).


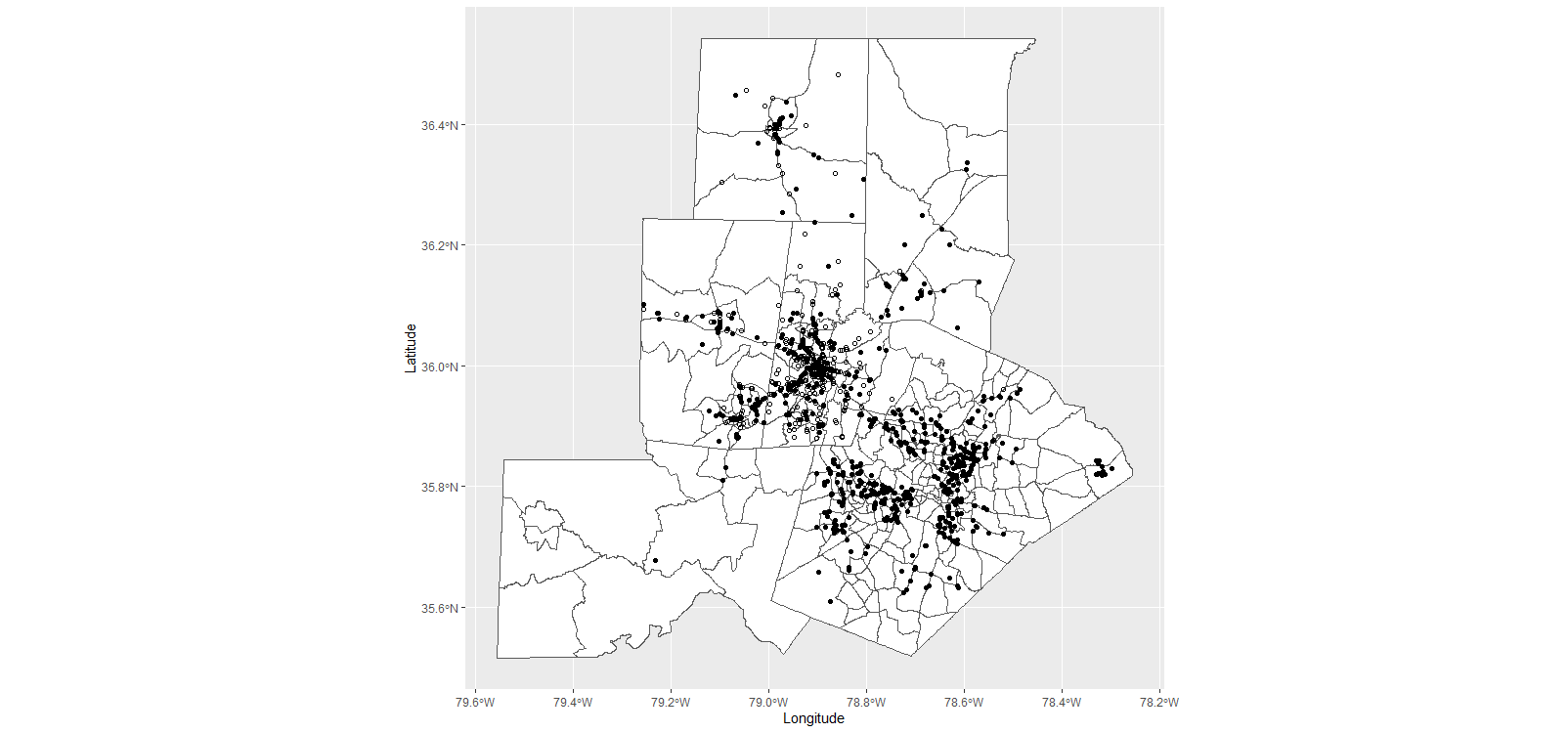


**Note:** Black circles denote TROs that existed both before and after the policy was enacted. Transparent circles denote TROs that only existed before but not after the policy was enacted.

1. https://default.sfplanning.org/publications_reports/SF_NGBD_SocioEconomic_Profiles/2012-2016_ACS_Profile_SupeDistricts_Final.pdf [↑](#footnote-ref-1)
